# Intimate partner violence and associated factors among adult women: a population-based study

**DOI:** 10.11606/s1518-8787.2026060007176

**Published:** 2026-06-12

**Authors:** Camila Simon, Juvenal Soares Dias da Costa, Maria Teresa Anselmo Olinto, Fernanda Souza de Bairros, Tonantzin Ribeiro Gonçalves

**Affiliations:** I Prefeitura Municipal de Porto Alegre. Hospital de Pronto-Socorro de Porto Alegre. Porto Alegre, RS, Brasil; II Universidade Federal de Pelotas. Faculdade de Medicina. Departamento de Medicina Social. Pelotas, RS, Brasil; III Universidade Federal do Rio Grande do Sul. Programa de Pós-Graduação em Alimentação, Nutrição e Saúde. Porto Alegre, RS, Brasil; IV Universidade Federal do Rio Grande do Sul. Programa de Pós-Graduação em Ciências Médicas. Porto Alegre, RS, Brasil; V Universidade Federal do Rio Grande do Sul. Programa de Pós-Graduação em Saúde Coletiva. Porto Alegre, RS, Brasil; VI Universidade Federal de Ciências da Saúde de Porto Alegre. Programa de Pós-Graduação em Psicologia e Saúde. Porto Alegre, RS, Brasil

**Keywords:** Intimate Partner Violence, Violence Against Women, Population Studies in Public Health, Brazil

## Abstract

**OBJECTIVE:**

The study investigated the lifetime prevalence of intimate partner violence among women aged 20 to 69 years residing in the urban area of São Leopoldo (RS) and associated factors.

**METHODS:**

This was a population-based cross-sectional study in which 1,113 women were interviewed. The outcome was self-reported lifetime intimate partner violence, and sociodemographic, physical and mental health, and behavioral variables were assessed. A stratified analysis by age group was performed.

**RESULTS:**

The prevalence of intimate partner violence in the sample was 17.9% (95%CI 15.6–20.1). In the adjusted analysis, the outcome was associated with lower socioeconomic status, a history of sexually transmitted infections, and the presence of common mental disorders. In the stratified analysis, women aged 20–39 years had a 13.7% (95%CI 10.5–16.8) lifetime prevalence of intimate partner violence, with nearly three times the likelihood among those with common mental disorders. Among women aged 40 years or older, the prevalence was 20.7% (95%CI 17.6–23.8), with those in the lowest socioeconomic class, who were single, divorced, or widowed, who self-reported sexually transmitted infections, and who had common mental disorders being more likely to experience the outcome. Secondary intersectional analyses revealed a higher prevalence of intimate partner violence among Black or Brown women with low educational attainment (PR = 1.67; 95%CI 1.14–2.45) and White women with low educational attainment (PR = 1.58; 95%CI 1.14–2.19), compared to white women with higher educational attainment.

**CONCLUSIONS:**

The study demonstrated a high lifetime prevalence of intimate partner violence, reinforcing the need for public policies that improve women’s socioeconomic conditions, as well as integrated gender-based violence prevention programs that incorporate mental health care in the support provided to victims.

## INTRODUCTION

Violence against women is defined by the United Nations as “any act of gender-based violence that results in, or is likely to result in, physical, sexual, or mental harm or suffering to women, including threats of such acts, coercion, or arbitrary deprivation of liberty, whether occurring in public or private life”^1^. Violence against women is a public health issue that intersects with the defense of human rights, and cases of such violence, in addition to being widespread, demonstrate a serious and recurring nature in our society^2^. In most cases, physical, psychological, and sexual violence overlap, and intimate partners are the most frequent perpetrators of violence against women^2^.

Intimate partner violence (IPV) consists of any and all violent behavior committed by a person with whom the victim has or has had (former partners) an intimate relationship, whether or not perpetrated within the domestic unit and regardless of cohabitation^3^. It encompasses physical, psychological, or sexual abuse, which may include assaults, punishments, deprivation of liberty, threats, sexual offenses, controlling behavior, humiliations, degrading acts, and the like. The most common form of violence perceived and reported by women is physical violence, and the most serious consequence of IPV is femicide, constituting the tragic culmination of the cycle of gender-based violence in intimate relationships^5^.

Data from the World Health Organization indicate that one in three women (30%) worldwide has experienced physical and/or sexual violence at the hands of an intimate partner, and up to 38% of all murders of women are committed by them^1^. Specifically in the Americas, 25% of women reported experiencing IPV at some point in their lives. Among women aged 15 to 49, 27% of those who have ever been in a relationship were subjected to some form of violence by their partners in 2018^6^.

It is worth noting that, beyond these alarming figures, the problem may be even more severe, given that cases are underreported. Only a fraction of women who are victims of IPV manage to overcome the barriers of fear, psychological and/or financial dependence on the perpetrator, and the social constructs related to gender and male dominance, to file a report^7^.

IPV has a considerable impact on women’s physical and mental health. Although physical stigmas, such as fractures, lacerations, bruises, and other injuries, are more visible, one must also consider the repercussions that violence has on sexual health, including sexually transmitted infections (STIs) and unwanted pregnancies^8^. Furthermore, equally or even more significant are the consequences for mental health, which include an increased risk of depression, anxiety, post-traumatic stress disorder, substance abuse, and suicidal behaviors^8^. Therefore, in addition to violating human rights, intimate partner violence against women increases the demand for health care and results in economic losses for society.

Although there are several Brazilian studies on the subject, they remain scarce and are concentrated in the southeastern and northeastern regions of the country^9^. Furthermore, most studies focus on the sociodemographic profile of victims and the care they receive^8, 12^. Among the few population-based studies on the topic, the study using data from the 2019 Brazilian National Health Survey, which assessed 34,334 women^15^, and the population-based study conducted in a rural area of Rio Grande do Sul, involving 971 women^16^, stand out. Finally, only one Spanish study and one Australian study were identified that addressed IPV from a generational perspective among women^17,18^, which represents a distinguishing feature of this research. Given this, the present study aims to investigate the prevalence of IPV among women aged 20 to 69 years, residing in the urban area of the municipality of São Leopoldo (RS), and the associated factors.

## METHOD

This is a cross-sectional, population-based epidemiological study with a representative sample of adult women residing in the urban area of the municipality of São Leopoldo (RS) in 2015. The broader study from which this study was derived included women aged 20 to 69 years to account for the upper age limit for adult samples, with the aim of addressing relevant health outcomes such as mammography screening and medication use.

The municipality is located in the Vale do Rio dos Sinos, in the Porto Alegre Metropolitan Region, 31 km from the capital. It has a population of 217,409 inhabitants, according to the latest Census, of whom approximately 52% are women. Among women, approximately 65% are in the 20–69 age group^19^.

The sample size for the larger study was estimated based on a calculation that would allow the identification of a prevalence ratio of 2.0 at a 95% confidence level and 80% statistical power, maintaining a non-exposed:exposed ratio of 1:2 for the education variable (according to the distribution in the municipality in 2010 and the 6% prevalence of the least frequent outcome assessed—delayed cytopathological examination). The sample size was estimated at 905 women, with an additional 10% added to account for potential losses and/or refusals and 15% to control for confounding factors, totaling 1,130 women.

Considering the average number of residents per household and the age group of interest, 1,451 households were visited, based on cluster sampling. Forty census tracts were randomly selected from among the 270 in the urban area of São Leopoldo. In each tract, the block and the starting corner for data collection were randomly defined, followed by the alternate selection of households (every other house) until the required number per tract was reached. Further details are available in Osmari et al.^20^ In the present analysis, those who reported exclusive sexual relations with women were excluded due to low frequency (n = 12) and because IPV among same-sex couples involves distinct social dynamics not addressed by the survey instrument^21^.

The outcome of this study was “intimate partner violence,” a dichotomous variable assessed by the question: “Has a partner ever assaulted you in your lifetime?” Independent variables included sociodemographic, behavioral, sexual, reproductive, and mental health factors.

The sociodemographic variables were: age (20–29, 30–39, 40–49, 50–59, and 60–69 years), skin color (white, brown/black, and other), marital status (married or in a union, single, divorced, or widowed), socioeconomic class (A+B, C, and D+E; according to the Brazilian Economic Classification Criteria - ABEP, 2015), and education level (0–4, 5–10, 11–14, and ≥ 15 years of schooling).

Regarding behavioral, sexual, reproductive, and mental health variables, the following were considered: age at first pregnancy (≤ 17 / ≥ 18 years), number of pregnancies (none, one, two, three, or more), self-reported induced abortion (yes/no), history of STIs (yes/no), number of medical visits in the past year (none/one/two or more), smoking (never smoked/former smoker/smoker), excessive alcohol use in the past 30 days (yes/no; ≥ 30g/day of ethanol^22^ ), lifetime illicit drug use (yes/no), and presence of common mental disorders (CMD) (yes/no; score ≥ 7 on the Self- Questionnaire - SRQ-20). The SRQ-20 consists of 20 dichotomous (yes/no) questions designed to screen for CMDs^23^.

Data collection was conducted using a standardized, pre-coded, and pre-tested questionnaire administered by interviewers. Questions regarding abortion, STIs, illicit drug use (marijuana, *crack*, cocaine, and others), and VPI were included in a self-administered questionnaire, which could be answered and sealed by the participant herself, then deposited in a box. In a few cases, where the woman was illiterate (n = 3) or unable to complete the self-administered questionnaire on her own—for example, due to visual impairment—the interviewers assisted in filling it out. The interviews were conducted individually by 18 trained interviewers between February and October 2015, following strict standardization protocols. If necessary, the interviewers returned to the home at a time more convenient for the woman to ensure privacy during the interview.

Data were entered in duplicate using EpiData, and analyses were performed using SPSS v.25 and Stata v.15. Associations between the outcome and the independent variables were tested using Pearson’s chi-square test and the linear association test. Crude and adjusted prevalence ratios, with 95% confidence interval (95%CI), were estimated using Poisson regression with robust variance. Variables with p < 0.20 in the bivariate analysis were included in the adjusted analysis, structured into two blocks defined by a prior conceptual model^24^: (1) sociodemographic; (2) sexual, reproductive, behavioral, and mental health. A stratified analysis was performed by age groups (20–39 and ≥ 40 years). This stratification was chosen based on the sample distribution, as more than 40% of the women were 39 years old or younger (born after 1975), and to ensure statistical stability in the multivariate analyses with only two age categories. Furthermore, this stratification sought to identify women with potentially distinct trajectories regarding the sociocultural patterns of gender relations and exposure to IPV.

Furthermore, an exploratory and secondary intersectional analysis^26^ was conducted on the prevalence of IPV according to combinations of race/color and education level, adjusted for age and marital status. Due to low frequency, this analysis did not include Asian and Indigenous women, with the category dichotomized into Black/Brown and White. Furthermore, the education variable, also considered dichotomously, was chosen for its greater potential to describe inequalities among women, since the economic class variable includes the education level of the head of the household (ABEP, 2015) in its calculation, which, in some cases, may not be the woman herself. Thus, four intersectional groups were considered: 1) white women with ≥ 11 years of schooling (reference group); 2) Black/Brown women with ≥ 11 years; 3) white women with ≤ 10 years; 4) Black/Brown women with ≤ 10 years of schooling.

This study was approved by the Ethics and Research Committee of the Universidade do Vale do Rio dos Sinos.

## RESULTS

A total of 1,281 women were visited, and 1,128 were interviewed, with 153 (11.9%) classified as losses and refusals. Among the interviewees, data from 1,113 women who answered the question regarding the outcome were considered. Most were aged 40–49 years (24.4%), self-identified as white (74.6%), 58% had up to 10 years of schooling, and 53% belonged to socioeconomic class C (Table 1). Ultimately, 199 (17.9%; 95%CI 15.6–20.1) reported having experienced IPV at some point in their lives.

**Table 1:** Prevalence of intimate partner violence in lifetime, according to sociodemographic, sexual, reproductive, behavioral, and mental health variables in adult women (n = 1,113).

Variables	n (%)	IPV n (%)	(95%CI)
Ages			
20–29 years	213 (19)	29 (13)	(9–18)
30–39 years	240 (22)	33 (14)	(9–18)
40–49 years	272 (24)	63 (23)	(18–28)
50–59 years	226 (20)	48 (21)	(16–26)
60–69 years	162 (15)	26 (16)	(10–21)
Education			
≥ 15 years	108 (10)	12 (11)	(5–17)
11–14 years	359 (32)	45 (12)	(9–16)
5–10 years	444 (40)	95 (21)	(17–25)
0–4 years	200 (18)	47 (23)	17–29
Skin color			
White	830 (75)	146 (18)	(15–20)
Yellow, brown, indigenous, black	283 (25)	53 (19)	(14–23)
Economic class			
A and B	386 (35)	39 (10)	(7–13)
C	587 (53)	120 (20)	(17–23)
D and E	134 (12)	40 (30)	(22–37)
Marital status			
Married/in a stable relationship	402 (64)	113 (16)	(17–25)
Single/divorced/widowed	711 (36)	86 (21)	(13–18)
Number of pregnancies			
None	170 (15)	14 (8)	(4–12)
One	251 (23)	33 (13)	(9–17)
Two	265 (24)	39 (15)	(10–19)
Three or more	427 (38)	113 (26)	(22–30)
Age at first pregnancy			
≥ 18 years	716 (76)	126 (18)	(14–20)
Up to 17 years	224 (24)	57 (25)	(19–31)
Lifetime abortion			
No	1014 (91)	172 (17)	(14–19)
Yes	95 (9)	27 (28)	(19–37)
STIs in life			
No	1032 (93)	175 (17)	(14–19)
Yes	79 (7)	24 (30)	(20–40)
Number of visits			
None	174 (15)	24 (14	(8–19)
One	176 (16)	31 (18)	(12–23)
Two or more	762 (68)	144 (19)	(16–21)
Excessive alcohol consumption			
No	1075 (97)	190 (18)	(15–20)
Yes	32 (3)	7 (22)	(6–37)
Smoking			
Non-smoker	653 (59)	91 (14)	(11–16)
Former smoker	251 (23)	56 (22)	(17–27)
Current smoker	203 (18)	52 (26)	(19–31)
Lifetime illicit drug use			
No	983 (88)	172 (17)	(15–20)
Yes	130 (12)	27 (21)	(13–27)
Common mental disorder			
< 7	670 (60)	84 (12)	(10–15)
≥ 7	443 (40)	115 (26)	(22–30)

95%CI: 95% confidence interval; IPV: intimate partner violence; STIs: sexually transmitted infections.

In the unadjusted analysis, the following variables were associated with the outcome of PID: lower socioeconomic status, belonging to socioeconomic classes C, D, and E, being married or in a stable relationship, having had an STI in one’s lifetime, age at first pregnancy up to 17 years, being a current or former smoker, and the presence of CMD (Table 2). It was found that, after adjusted analysis, lower socioeconomic class, having a partner, having had an STI in one’s lifetime, and the presence of CMD continued to be associated with the outcome. Women in economic classes D/E who reported having had an STI were approximately twice as likely to experience the outcome, while women with CMD were 50% more likely to experience IPV compared to those without CMD.

**Table 2:** Crude and adjusted prevalence ratios of lifetime intimate partner violence, according to sociodemographic, sexual, reproductive, behavioral, and mental health variables in adult women (n = 1,113).

Variable	Crude analysis		Adjusted analysis	
	PR (95%CI)	p	PR (95%CI)	p
Age^a^		0.063		
20–29 years	1.00			
30–39 years	1.01 (0.63–1.60)			
40–49 years	1.70 (1.14–2.54)			
50–59 years	1.56 (1.02–2.38)			
60–69 years	1.18 (0.72–1.92)			
Education level		< 0.001		
≥ 15 years	1.00			
11–14 years	1.13 (0.62–2.05)			
5–10 years	1.92 (1.10–3.38)			
0–4 years	2.11 (1.17–3.81)			
Skin color^a^		0.665		
White	1.00			
Yellow, brown, indigenous, black	1.06 (0.80–1.41)			
Economic class^a^		**< 0.001**		**0.001**
A and B	**1.00**		**1.00**	
C	**2.02 (1.44–2.84)**		**1.62 (1.15–2.27)**	
D and E	**2.95 (1.99–4.39)**		**1.94 (1.29–2.93)**	
Marital status		**0.021**		0.059
Married/in a stable relationship	**1.00**		1.00	
Single/divorced/widowed	**1.35 (1.04–1.73)**		1.29 (1.00–1.66)	
Number of pregnancies^b^		< 0.001		< 0.001
None	1.00		1.00	
One	1.60 (0.88–2.89)		1.48 (0.81–2.71)	
Two	1.79 (1.00–3.19)		1.76 (0.98–3.18)	
Three or more	3.21 (1.90–5.44)		2.40 (1.39–4.16)	
Age at first pregnancy^b^		0.008		
≥ 18 years	1.00			
Up to 17 years	1.45 (1.10–1.90)			
Lifetime abortion^b^		0.004		
No	1.00			
Yes	1.68 (1.18–2.37)			
STIs in lifetime^b^		**0.002**		**0.002**
No	**1.00**		**1.00**	
Yes	**1.79 (1.25–2.57)**		**1.70 (1.19–2.42)**	
Number of visits^b^		0.126		0.119
None	1.00		1.00	
One	1.28 (0.78–2.08)		1.54 (0.95–2.49)	
Two or more	1.37 (0.92–2.04)		1.41 (0.95–2.09)	
Excessive alcohol consumption^b^		0.531		
No	1.00			
Yes	1.24 (0.63–2.41)			
Smoking^b^		**< 0.001**		0.031
Non-smoker	**1.00**		1.00	
Former smoker	**1.60 (1.19–2.16)**		1.30 (0.97–1.75)	
Current smoker	**1.84 (1.36–2.49)**		1.33 (0.98–1.81)	
Lifetime illicit drug use^b^		0.354		
No	1.00			
Yes	1.19 (0.83–1.71)			
Common mental disorder^b^		**< 0.001**		**< 0.001**
< 7	**1.00**		**1.00**	
≥ 7	**2.07 (1.60–2.67)**		**1.71 (1.32–2.21)**	

95%CI: 95% confidence interval; PR: prevalence ratio; STIs: sexually transmitted infections.

Note: values in bold: p < 0.05 and 95%CI with effect.

^a^ Block of sociodemographic variables.

^b^ Block of behavioral, sexual, reproductive, and mental health variables. Each variable was adjusted for the others at the same level or at a higher level in a hierarchical causality model. Only variables associated with the e outcome with p < 0.20 in the unadjusted model were subsequently included and retained in the final adjusted multivariate model.

The analysis stratified by age groups aimed to examine possible generational aspects related to the outcome. Among women aged 20–39 years, the lifetime prevalence of VPI was 13.7% (95%CI 10.5–16.8), and for those aged 40 years or older, it was 20.7% (95%CI 17.6–23.8). Women aged 20–39 who had had three or more pregnancies, had undergone an abortion, were current or former smokers, and had CMD had higher prevalences of the outcome in the crude analysis (Table 3). In the adjusted analysis, only the presence of CMD remained associated, with these women being 2.33 times more likely to have experienced VPI.

**Table 3:** Crude and adjusted prevalence ratios of lifetime intimate partner violence, according to sociodemographic, sexual, reproductive, behavioral, and mental health variables in adult women, stratified by age groups: 20–39 years, 40 years or older (n = 1,113).

Variables	20 to 39 years				40 years or older			
	Crude Analysis		Adjusted Analysis		Crude Analysis		Adjusted Analysis	
	PR (95%CI)	p	PR (95%CI)	p	PR (95%CI)	p	PR (95%CI)	p
Education level^a^		0.014		0.043		0.017		
≥ 15 years	1.00		1.00		1.00			
11 to 14 years	1.07 (0.42–2.73)		1.06 (0.42–2.63)		1.23 (0.56–2.68)			
5 to 10 years	2.19 (0.90–5.33)		1.94 (0.80–4.70)		1.69 (0.82–3.48)			
0 to 4 years	1.63 (0.47–5.59)		1.46 (0.43–4.92)		1.88 (0.90–3.93)			
Skin color^a^		0.753				0.430		
White	1.00				1.00			
Yellow, brown, indigenous, black	0.92 (0.53–1.58)				1.14 (0.82–1.59)			
Economic class^a^		0.038				**< 0.001**		**< 0.001**
A and B	1.00				**1.00**		**1.00**	
C	1.64 (0.93–2.89)				**2.25 (1.48–3.44)**		**1.76 (1.15–2.69)**	
D and E	1.96 (0.94–4.11)				**3.55 (2.21–5.69)**		**2.30 (1.42–3.75)**	
Marital status		0.199				**< 0.001**		**0.009**
Married/in a stable relationship	1.00				**1.00**		**1.00**	
Single/divorced/widowed	0.71 (0.43–1.19)				**1.76 (1.31–2.36)**		**1.46 (1.09–1.95)**	
Number of pregnancies^b^		**0.002**				< 0.001		0.008
None	**1.00**				1.00		1.00	
One	**2.10 (1.00–4.40)**				0.89 (0.33–2.41)		0.84 (0.31–2.25)	
Two	**1.72 (0.71–4.15)**				1.29 (0.53–3.14)		1.30 (0.54–3.14)	
Three or more	**3.18 (1.53–6.61)**				2.27 (0.98–5.27)		1.71 (0.73–3.97)	
Age at first pregnancy^b^		0.223				**0.003**		
≥ 18 years	1.00				**1.00**			
Up to 17 years	1.36 (0.83–2.24)				**1.64 (1.18–2.28)**			
Lifetime abortion^b^		**0.024**				0.081		
No	**1.00**				1.00			
Yes	**2.17 (1.10–4.27)**				1.43 (0.96–2.15)			
STIs in lifetime^b^		0.183				**0.001**		**0.001**
No	1.00				**1.00**		**1.00**	
Yes	1.57 (0.81–3.06)				**2.04 (1.35–3.08)**		**1.97 (1.30–2.97)**	
Number of visits^b^		0.409		0.193		0.386		
None	1.00		1.00		1.00			
One	1.06 (0.46–2.43)		1.20 (0.53–2.73)		1.30 (0.70–2.41)			
Two or more	1.27 (0.68–2.37)		1.44 (0.79–2.65)		1.30 (0.77–2.20)			
Excessive alcohol consumption^b^		0.594				**0.018**		
No	1.00				**1.00**			
Yes	0.69 (0.18–2.65)				**2.24 (1.15–4.35)**			
Smoking^b^		**0.002**				**0.006**		0.203
Non-smoker	**1.00**				**1.00**		1.00	
Former smoker	**1.95 (1.09–3.46)**				**1.35 (0.95–1.93)**		1.15 (0.83–1.62)	
Smoker	**2.12 (1.24–3.63)**				**1.64 (1.14–2.35)**		1.25 (0.88–1.77)	
Lifetime illicit drug use^b^		0.159				0.267		
No	1.00				1.00			
Yes	1.44 (0.87–2.40)				1.36 (0.79–2.36)			
Common mental disorder^b^		**< 0.001**		**0.001**		**< 0.001**		**0.006**
< 7	**1.00**		**1.00**		**1.00**		**1.00**	
≥ 7	**2.59 (1.59–4.20)**		**2.33 (1.41–3.87)**		**1.90 (1.42–2.57)**		**1.50 (1.12–2.01)**	

95%CI: 95% confidence interval; PR: prevalence ratio; STIs: sexually transmitted infections.

Note: values in bold: p < 0.05 and 95%CI with effect.

^a^ Block of sociodemographic variables.

^b^ Block of behavioral, sexual, reproductive, and mental health variables. Each variable was adjusted for the others at the same level or at a higher level in a hierarchical causal model. Only variables associated with the outcome with p < 0.20 in the unadjusted model were subsequently included and retained in the final multivariate adjusted model.

Among women aged 40 years or older, the occurrence of the outcome was associated with lower socioeconomic status, lower educational attainment, being single, divorced, or widowed, a higher number of pregnancies, being younger at first pregnancy, having had an STI in their lifetime, reporting an abortion, being a current or former smoker, excessive alcohol consumption, and the presence of CMD (Table 3). In the adjusted analysis, the variables economic class, marital status, number of pregnancies, STIs, smoking, and presence of CMD remained significant for the outcome.

It was found that, among women aged 40 years or older in socioeconomic classes D and E, the probability of IPV was more than twice as high compared to women in class A. Furthermore, among these women, those who were single, divorced, or widowed were 1.45 times more likely to experience IPV. A history of STIs nearly doubled the probability of VPI, and those with CMD had a prevalence ratio of 1.50 (Table 3).

Secondary analyses explored the association between VPI and the intersection of race/color and education categories. For comparison purposes, the Figure shows the crude prevalence ratios of VPI for education and race/color separately and the adjusted prevalences for the intersection categories. The adjusted prevalence ratios for the outcome were 1.67 times higher (95%CI 1.14–2.45) among Black or Brown women with 10 years or less of schooling (n = 178) compared to White women with 11 years or more of schooling (index group; n = 376). White women with lower educational s (n = 453) were also more likely to have a lifetime history of VPI (PR = 1.58; 95%CI 1.14–2.19), while black women with higher education (n = 81) showed a lower prevalence of the outcome (PR = 0.67; 95%CI 0.31–1.42), although this was not statistically significant.

**Figure:**
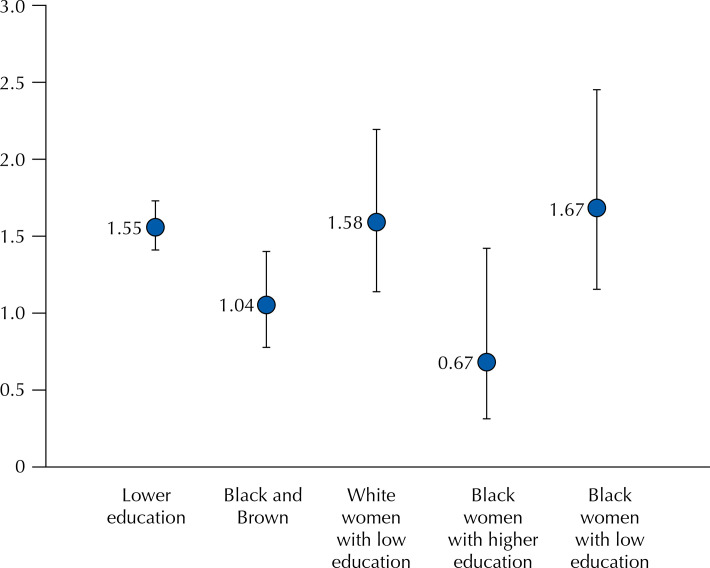
Crude (education and race/color) and adjusted (intersected categories) prevalence ratios of lifetime intimate partner violence (n = 1,088 ). Note: intersectional categories were adjusted for age and marital status.

## DISCUSSION

In this study, a high lifetime prevalence of IPV was observed among adult women residing in the urban area of a municipality in southern Brazil, with the highest frequency among those aged 40 years or older. IPV was associated with lower socioeconomic status, marital status (being single, divorced, or widowed), a history of STIs, and the presence of CMD, revealing the interaction between social, sexual, and psychological vulnerabilities. When stratified by age group, distinct patterns of association emerged: among women aged 20 to 39, IPV was most strongly associated with CMD, whereas among those aged 40 or older, a higher probability of IPV was observed among those in lower socioeconomic classes, with a history of STIs, and who were single, widowed, or divorced. Furthermore, Black or brown women with lower levels of education were more likely to experience IPV, pointing to the intersectional influence of structural racism and inequalities in the production of gender-based violence.

The prevalence found was similar to that of a population-based study conducted in rural Rio Grande do Sul, where 17.2% of women reported psychological violence at least once in their lives^16^. On the other hand, it was lower than that observed in a multicenter study involving Brazil, which reported more than 45% of psychological, physical, or sexual violence among urban women^2^, as well as that recorded in Maringá (56.0%)^27^. However, it was higher than the prevalence described in the 2019 National Health Survey (7.6% in the past 12 months)^28^.

Global estimates from 2000 to 2018 reported that 27% of women aged 15 to 49 had been victims of physical or sexual violence by an intimate partner at least once in their lifetime^1^. In Latin America and the Caribbean, the lifetime prevalence of IPV was estimated at 25%, and in Brazil, at 23%^1^ – figures close to those of this study. A meta-analysis reported a prevalence of 37.3% for any type of IPV in a lifetime and 24.2% in the past year, with psychological violence being the most common^29^.

The prevalence rates of IPV vary widely across national and international studies, making direct comparisons difficult. Part of these differences may be related to the type of IPV assessed and the recall period used. While the present study investigated lifetime IPV through direct self-report, other surveys applied standardized instruments that distinguish between types of violence and different periods of occurrence. Thus, the prevalence measured here may be underestimated, as many women still do not recognize psychological violence, especially in its more subtle forms, or normalize it^8^. Furthermore, variations in urban and rural contexts, in the age composition of the samples, and in data collection methods may explain part of the discrepancies observed in the recorded prevalence rates. Nevertheless, the use of a ballot box—a strategy rarely employed in Brazilian surveys on violence—may have helped reduce social desirability bias and enhance the validity of self-reported information.

In this study, IPV was associated with lower socioeconomic status, being single, divorced, or widowed, reporting an STI, and the presence of CMD. IPV results from the interaction of factors at the individual, family, community, and societal levels^1^. Limited access to employment and income are recognized as factors associated with IPV^1,9,11,12,15^ and partly reflect socioeconomic status, which in this study tripled the likelihood of IPV. A similar finding was reported in a national survey in South Africa, where women from households with higher wealth indices had a lower incidence of sexual violence^30^. Thus, economic inequality—structural in the Global South—is at the root of and contributes to the perpetuation of IPV^31^. Poorer women, with lower levels of education and access to paid employment, tend to be economically dependent on the perpetrator, which makes it difficult to break the cycle of violence^12,30^.

Lifetime occurrence of STIs was also associated with IPV. Although few Brazilian studies address sexual factors and health behaviors linked to IPV, our findings align with reviews indicating an association with STIs, unwanted pregnancies, abortions, and gynecological symptoms among women who have experienced sexual violence^32^. Another review pointed to a high prevalence of pelvic and gynecological pain and STIs among victims of IPV in different countries^3^.

An association was observed between the presence of CMD and VPI, with women who reported symptoms of CMD being nearly twice as likely to report VPI. In the total sample, 39.8% of women showed indicators of CMD, a percentage that increased to 57.8% among those who reported VPI. The same pattern was observed in the analysis stratified by age group, with a higher prevalence among women aged 39 and under. These findings are consistent with the literature, including international studies, which have demonstrated a strong association between IPV and psychological distress^29,33^.

Evidence indicates that being a victim of IPV increases the risk of mental disorders, and in some cases, this relationship is bidirectional, given the presence of common risk factors^35^. Reviews of longitudinal studies suggest that depression may precede domestic violence, while experiencing violence increases the risk of depression even in previously healthy women^34^. Another Australian longitudinal study with a generational focus identified poorer mental health associated with IPV across all ages, with a greater impact in old age, and showed that women with poorer mental health in youth were more likely to experience IPV throughout their lives^17^.

In Brazil, a prospective cohort study of 390 women showed a 44.6% incidence of major depressive disorder among victims of violence in the previous 12 months and a 43.4% incidence among those who reported violence in the past seven years^36^. Meta-analyses reinforce this association, indicating a threefold increase in the likelihood of depressive disorders, a fourfold increase for anxiety disorders, and up to a sevenfold increase for post-traumatic stress disorder among women who are victims of violence^37^. Another meta-analysis confirmed the association between IPV and depression, PTSD, and suicide risk^29^. In the United Kingdom, a study showed that 58.4% of women who had attempted suicide in the previous year had experienced IPV at some point in their lives^38^.

Several studies, using different methodologies, have pointed to an association between IPV and race/ethnicity ^9, 10 15^, which differs from the findings of the present study. Our secondary analyses suggest that the intersection of race/ethnicity and educational attainment offers a more adequate explanation for social inequalities related to IPV, as it reflects structural racism and the historical exclusion of Black people^39^. In the study, Black or Brown women with lower r educational attainment were more likely to have VPI than those in the other intersectional categories (White women with low or high educational attainment and Black/Brown women with high educational attainment). Due to limited statistical power, this hypothesis should be tested in future studies with sampling designed to capture these social intersections.

We opted for age-stratified analysis to capture possible generational influences on the association between variables and VPI—an approach still rarely adopted in studies on this topic. Among women aged 20 to 39, VPI was associated with the presence of CMD; among those aged 40 or older, there was an association with lower socioeconomic status, marital status (single, widowed, or divorced), and reports of STIs and CMD.

Contrary to the results of this study, which indicated higher prevalences among older women, data from IPV notifications in Brazil (2011–2017) found higher incidences of IPV among women aged 20 to 39^8^. Meanwhile, a Spanish national survey of 8,935 women, which stratified the prevalence of IPV, associated factors, and impacts of IPV by age (16–29, 30–49, and ≥ 50 years), revealed that 15.6% had experienced some form of IPV in the previous 12 months, with higher rates among younger women—which also contradicts our findings^18^. Nevertheless, it is plausible to assume that exposure to this outcome increases with advancing age, while younger women may recognize, report, or seek help more easily, and are therefore more frequently reported. Furthermore, methodological differences between studies that assessed the age group, and the present study may explain this discrepancy.

The Spanish study also associated economic vulnerability with a higher incidence of IPV among women aged 30 to 49^18^, as in the present study, since, among women aged 40 or older, the odds of IPV were twice as high as among women from lower socioeconomic classes. Regarding marital status, a higher probability of IPV was observed among single, divorced, or widowed women in this age group, but not among younger women. Previous cross-sectional studies had already pointed to a similar trend^40,41^, although without stratifying by life stage. One hypothesis for our findings is that older women may have experienced abusive relationships throughout their lives and, once they were more established, with adult children, managed to break the cycle of violence. Among younger women, however, the lower prevalence of IPV may be explained by shorter exposure.

When addressing IPV, a recurring limitation in studies involves stigma, shame, and guilt stemming from the hegemonic patriarchal culture^7,10^. Thus, despite the high prevalence found, the data may be underestimated, since many women conceal or have difficulty acknowledging the violence they have suffered—which may have been mitigated in this study by the comprehensive nature of the outcome assessment. Although population-based screening for IPV was facilitated by assessing the outcome through a single question, the sensitivity of the measurement may have been reduced for the different types of violence, attenuating the observed associations. Another limitation, inherent to the cross-sectional design, is the inability to determine whether IPV is a cause or a consequence of the associated factors. However, the study was conducted with methodological rigor, including the use of a ballot box to ensure the authenticity of responses and guarantee privacy during interviews. Furthermore, although the findings reflect the situation of adult women residing in São Leopoldo in 2015, it is believed that they provide detailed data that can inform actions to monitor violence against women.

## CONCLUSION

Collectively, the findings indicate the need for intersectoral public policies aimed at preventing and addressing IPV that integrate health, social assistance, and justice initiatives, with special attention to women in greater economic vulnerability and older age groups. We suggest the importance of strengthening strategies that link mental health care with improvements in socioeconomic conditions and social protection, as advocated by the National Policy to Combat Violence against Women^42^. Thus, the results can support the planning of continuing education initiatives and the ongoing training of health professionals from the perspective of the social complexity of the phenomenon, considering that health services may be sought for mental health issues that underline or mask situations of violence. It is understood that strengthening mental health matrixing, promotion, and prevention actions within the health system would not only facilitate the identification and management of situations of violence but also the development of strategies to prevent and mitigate the psychological repercussions of gender-based violence in the territories covered by primary health care.

Based on the generational patterns observed, preventive approaches should consider the different stages of the life cycle and the unfolding of vulnerability trajectories. In other words, among younger women, educational initiatives on abusive relationships and mental health may be more effective, as well as greater attention to signs of psychological distress within the healthcare setting. For older women, it would be important to expand and strengthen labor market integration policies, as well as community and social support networks that facilitate the breaking of violent bonds, with an emphasis on those with greater socioeconomic vulnerability.

Finally, we reiterate the importance of conducting new studies on this topic across diverse contexts to improve public policies, thereby benefiting women with a history of IPV or those currently experiencing this serious situation. It is suggested that the monitoring of IPV, whether in population surveys or in public services, includes indicators sensitive to inequalities of gender, race, and generation, in order to guide more equitable prevention policies.

## Data Availability

The database related to this research is available upon request to the corresponding author.
