# Supplementary material for: Intimate partner violence and associated factors among adult women: a population-based study
Source: Rev Saude Publica. 2026 Jun 12;60:e17. doi: 10.11606/s1518-8787.2026060007176 (PMC13271208; doi:10.11606/s1518-8787.2026060007176)
Supplement: Translation

# Translation: Violência por parceiro íntimo e fatores associados entre mulheres adultas: um estudo de base populacional

**Authors:** Camila Simon, Juvenal Soares Dias da Costa, Maria Teresa Anselmo Olinto, Fernanda Souza de Bairros, Tonantzin Ribeiro Gonçalves

**Affiliations:** I Prefeitura Municipal de Porto Alegre. Hospital de Pronto-Socorro de Porto Alegre. Porto Alegre, RS, Brasil; II Universidade Federal de Pelotas. Faculdade de Medicina. Departamento de Medicina Social. Pelotas, RS, Brasil; III Universidade Federal do Rio Grande do Sul. Programa de Pós-Graduação em Alimentação, Nutrição e Saúde. Porto Alegre, RS, Brasil; IV Universidade Federal do Rio Grande do Sul. Programa de Pós-Graduação em Ciências Médicas. Porto Alegre, RS, Brasil; V Universidade Federal do Rio Grande do Sul. Programa de Pós-Graduação em Saúde Coletiva. Porto Alegre, RS, Brasil; VI Universidade Federal de Ciências da Saúde de Porto Alegre. Programa de Pós-Graduação em Psicologia e Saúde. Porto Alegre, RS, Brasil

**Keywords:** Violência por Parceiro Íntimo, Violência contra a Mulher, Estudos Populacionais em Saúde Pública, Brasil

## Abstract

**OBJETIVO:**

O estudo investigou a prevalência de violência por parceiro íntimo na vida em mulheres de 20 a 69 anos, residentes na zona urbana de São Leopoldo (RS) e os fatores associados.

**MÉTODOS:**

Trata-se de estudo transversal de base populacional, no qual foram entrevistadas 1.113 mulheres. O desfecho foi o autorrelato de violência por parceiro íntimo na vida e avaliou-se variáveis sociodemográficas, saúde física, mental e comportamentos. Realizou-se uma análise estratificada por geração etária.

**RESULTADOS:**

Verificou-se prevalência de 17,9% (IC95% 15,6–20,1) de violência por parceiro íntimo na amostra. Na análise ajustada, associaram-se ao desfecho a menor classe econômica, ter tido infecções sexualmente transmissíveis na vida e a presença de transtornos mentais comuns. Na análise estratificada, mulheres entre 20 e 39 anos tiveram 13,7% (IC95% 10,5–16,8) de violência por parceiro íntimo na vida, com quase três vezes mais probabilidade entre aquelas com transtornos mentais comuns. Já nas mulheres com 40 anos ou mais a prevalência foi de 20,7% (IC95% 17,6–23,8), sendo que aquelas com classe econômica mais baixa, solteiras, divorciadas ou viúvas, com autorrelato de infecções sexualmente transmissíveis e presença de transtornos mentais comuns tiveram mais probabilidade do desfecho. Análises interseccionais secundárias revelaram maior prevalência de violência por parceiro íntimo entre mulheres pretas ou pardas com baixa escolaridade (RP = 1,67; IC95% 1,14–2,45) e brancas com baixa escolaridade (RP = 1,58; IC95% 1,14–2,19), comparadas às brancas com maior escolaridade.

**CONCLUSÕES:**

O estudo demonstrou alta prevalência de violência por parceiro íntimo na vida, o que reforça a necessidade de políticas públicas que melhorem as condições socioeconômicas das mulheres, bem como programas integrados de prevenção à violência de gênero que incorporem cuidados em saúde mental no acolhimento às vítimas.

## INTRODUÇÃO

A violência contra as mulheres é definida pelas Nações Unidas como “qualquer ato de violência de gênero que resulte ou possa resultar em danos ou sofrimentos físicos, sexuais ou mentais para as mulheres, inclusive ameaças de tais atos, coação ou privação arbitrária de liberdade, seja em vida pública ou privada”^1^. A violência contra as mulheres é uma questão de saúde pública que perpassa a defesa dos direitos humanos e, cujos casos, além de alta magnitude, demonstram caráter grave e reiterado em nossa sociedade^2^. Na maioria das vezes, existe superposição da violência física, psicológica e sexual e são os parceiros íntimos os mais frequentes agressores das mulheres^2^.

A violência por parceiro íntimo (VPI) consiste em todo e qualquer comportamento violento cometido por pessoa com a qual a vítima tenha ou tenha tido (ex-parceiros) relação íntima de afeto, perpetrado ou não na unidade doméstica e independentemente de coabitação^3^. Compreende maus-tratos físicos, psíquicos ou sexuais que podem incluir agressões, castigos, privações de liberdade, ameaças, ofensas sexuais, comportamento controlador, humilhações, atos de degradação e afins. A forma mais comum de violência percebida e relatada pelas mulheres é a violência física e a consequência mais grave da VPI é o feminicídio, constituindo-se no trágico ápice do ciclo da violência de gênero na intimidade^5^.

Dados da Organização Mundial da Saúde indicam que uma em cada três mulheres (30%) em todo o mundo já sofreram violência física e/ou sexual por parte de parceiro íntimo e até 38% de todos os assassinatos de mulheres são cometidos por eles^1^. Especificamente no continente americano, 25% das mulheres relataram a experiência de VPI ao longo da vida. Entre mulheres de 15 a 49 anos, 27% daquelas que já estiveram em um relacionamento foram submetidas a alguma forma de violência por seus parceiros em 2018^6^.

Cabe destacar que, para além dos números alarmantes, o problema pode ser ainda mais grave, uma vez que existe subnotificação dos casos. Apenas uma parcela das mulheres vítimas de VPI consegue vencer a barreira do medo, da dependência psíquica e/ou financeira do agressor e da construção social relacionada ao gênero e à dominação masculina, para registrar a denúncia^7^.

A VPI impacta de forma considerável a saúde física e mental das mulheres. Ainda que estigmas físicos, tais como fraturas, lacerações, hematomas e outros traumas, sejam mais visíveis, há de que se considerar as repercussões que a violência tem na saúde sexual, com as infecções sexualmente transmissíveis (IST) e as gestações indesejadas^8^. Além disso, tão ou mais relevantes são as consequências na saúde mental, que incluem risco aumentado de depressão, ansiedade, transtorno de estresse pós-traumático, abuso de substâncias psicoativas e comportamentos suicidas^8^. Portanto, além de violar os direitos humanos, a violência contra as mulheres por parceiro íntimo aumenta a demanda de cuidados em saúde e resulta em prejuízos econômicos à sociedade.

Embora existam diversas pesquisas brasileiras sobre o tema, estas ainda são escassas e se concentram nas regiões sudeste e nordeste do país^9^. Além disso, a maioria das pesquisas se debruça sobre o perfil sociodemográfico e os atendimentos realizados às vítimas^8, 12^. Dentre os poucos estudos de base populacional sobre a temática, destaca-se o estudo com dados da Pesquisa Nacional de Saúde do Brasil – 2019, que avaliou 34.334 mulheres^15^ e a pesquisa populacional realizada em zona rural do Rio Grande do Sul, com 971 mulheres^16^. Por fim, foram localizados apenas um estudo espanhol e um estudo australiano que abordaram a VPI sob uma perspectiva geracional das mulheres^17,18^, o que representa um diferencial desta pesquisa. Diante disso, o presente estudo tem como objetivo investigar a prevalência de VPI em mulheres de 20 a 69 anos, residentes na zona urbana do município de São Leopoldo (RS) e os fatores associados.

## MÉTODO

Trata-se de um estudo epidemiológico transversal e de base populacional, com uma amostra representativa de mulheres adultas, residentes na área urbana do município de São Leopoldo (RS) em 2015. O estudo mais amplo, do qual derivou o presente estudo, incluiu mulheres de 20 a 69 anos a fim de considerar o limite superior para amostras de adultas, visando a contemplar desfechos relevantes em saúde como realização de mamografia e uso de medicamentos.

O município localiza-se no Vale do Rio dos Sinos, Região Metropolitana de Porto Alegre, distante 31 km da capital. Possui uma população de 217.409 habitantes, conforme o último Censo, sendo que, destes, cerca de 52% são mulheres. Entre as mulheres, em torno de 65% representam a faixa etária de 20 a 69 anos^19^.

A amostra do estudo maior foi estimada com base em cálculo que permitisse a identificação de uma razão de prevalência de 2,0 para um nível de confiança de 95% e poder estatístico de 80%, mantida a razão de não expostos:expostos de 1:2 para a variável escolaridade (conforme distribuição no município no ano de 2010 e a prevalência de 6% do desfecho menos frequente avaliado – exame citopatológico atrasado). A amostra foi estimada em 905 mulheres, tendo sido acrescentados 10% para eventuais perdas e/ou recusas e 15% para controle de fatores de confusão, totalizando 1.130 mulheres.

Considerando a média de moradores por domicílio e a faixa etária de interesse, foram visitados 1.451 domicílios, com base em amostragem por conglomerados. Sorteou-se 40 setores censitários entre os 270 da zona urbana de São Leopoldo. Em cada setor, definiu-se aleatoriamente o quarteirão e a esquina de início da coleta, seguindo-se a seleção alternada dos domicílios (a cada duas casas) até atingir o número necessário por setor. Mais detalhes estão disponíveis em Osmari et al.^20^ Na presente análise, foram excluídas aquelas que declararam relações sexuais exclusivas com mulheres, devido à baixa frequência (n = 12) e porque a VPI entre casais do mesmo sexo envolve dinâmicas sociais distintas não contempladas pelo instrumento da pesquisa^21^.

O desfecho do presente estudo foi a “agressão por parceiro íntimo”, variável dicotômica avaliada pela pergunta: “Alguma vez na vida, um companheiro seu agrediu você?” As variáveis independentes incluíram aspectos sociodemográficos, comportamentais, sexuais, reprodutivos e de saúde mental.

As variáveis sociodemográficas foram: idade (20–29, 30–39, 40–49, 50–59 e 60–69 anos), cor da pele (branca, parda/preta e outras), situação conjugal (casadas ou em união, solteiras, divorciadas ou viúvas), classe econômica (A+B, C e D+E; segundo o Critério de Classificação Econômica do Brasil - ABEP, 2015), escolaridade (0–4, 5–10, 11–14 e ≥ 15 anos de estudo).

Quanto às variáveis comportamentais, sexuais, reprodutivas e de saúde mental consideraram-se: idade da primeira gestação (≤ 17 / ≥ 18 anos), o número de gestações (nenhuma, uma, duas, três ou mais), autorrelato de aborto provocado (sim/não), história de IST (sim/não), número de consultas médicas no último ano (nenhuma/uma/duas ou mais), tabagismo (nunca fumou/ex-fumante/fumante), uso excessivo de álcool nos últimos 30 dias (sim/não; ≥ 30g/dia de etanol^22^), uso de drogas ilícitas na vida (sim/não) e presença de transtornos mentais comuns (TMC) (sim/não; pontuação ≥ 7 no *Self Reporting Questionnaire* - SRQ-20). O SRQ-20 é composto por 20 questões com respostas dicotômicas (sim/não), voltadas à triagem de TMC^23^.

A coleta de dados foi realizada por meio de questionário padronizado, pré-codificado e pré-testado, aplicado por entrevistadoras. Questões sobre aborto, IST, uso de drogas ilícitas (maconha, *crack*, cocaína e outras) e VPI foram incluídas em um questionário autoaplicado, que podia ser respondido e lacrado pela própria participante, sendo depositado em urna. Em poucos casos, em que a mulher era analfabeta (n = 3) ou não conseguia responder sozinha o questionário autoaplicado por exemplo, por deficiência visual, as entrevistadoras auxiliaram nesse preenchimento. As entrevistas foram conduzidas de modo individualizado por 18 entrevistadoras treinadas, entre fevereiro e outubro de 2015, seguindo protocolos rigorosos de padronização. Caso houvesse necessidade, as entrevistadoras retornavam ao domicílio em horário mais conveniente para a mulher, a fim de garantir a privacidade no momento da pesquisa.

A digitação foi feita em duplicata no programa EpiData, e as análises foram realizadas nos programas SPSS v.25 e Stata v.15. As associações do desfecho com as variáveis independentes foram testadas por meio do teste qui-quadrado de Pearson e do teste de associação linear. Estimaram-se razões de prevalência brutas e ajustadas, com intervalo de confiança de 95% (IC95%) por regressão de Poisson com variância robusta. Variáveis com p < 0,20 na análise bivariada foram incluídas na análise ajustada, estruturada em dois blocos definidos por modelo conceitual prévio^24^: (1) sociodemográficas; (2) sexuais, reprodutivas, comportamentais e de saúde mental. Realizou-se análise estratificada por faixas etárias (20–39 e ≥ 40 anos). A escolha dessa estratificação se baseou na distribuição amostral, pois mais de 40% das mulheres tinham até 39 anos (nascidas após 1975), e para garantir estabilidade estatística nas análises multivariadas com apenas duas categorias etárias. Além disso, com essa estratificação buscou-se identificar mulheres com trajetórias possivelmente distintas quanto aos padrões socioculturais das relações de gênero e de exposição à VPI.

Adicionalmente, foi conduzida uma análise interseccional^26^, exploratória e secundária, das razões de prevalência de VPI segundo combinações entre raça/cor e escolaridade, ajustada para idade e situação conjugal. Devido à baixa frequência, nessa análise, não foram incluídas mulheres amarelas e indígenas, dicotomizando-se para pretas/pardas e brancas. Além disso, a variável escolaridade, também considerada de modo dicotômico, foi escolhida pelo melhor potencial de descrição das desigualdades entre as mulheres, já que a variável classe econômica contempla em seu cálculo a escolaridade do chefe do lar (ABEP, 2015) que, em alguns casos, pode não ser a própria mulher. Assim, foram considerados quatro grupos interseccionais: 1) brancas com ≥ 11 anos de estudo (grupo referência); 2) pretas/pardas com ≥ 11 anos; 3) brancas com ≤ 10 anos; 4) pretas/pardas com ≤ 10 anos de escolaridade.

O presente estudo foi aprovado pelo Comitê de Ética e Pesquisa da Universidade do Vale do Rio dos Sinos.

## RESULTADOS

Foram visitadas 1.281 mulheres e 1.128 foram entrevistadas, sendo que 153 (11,9%) foram classificadas como perdas e recusas. Dentre as entrevistadas, foram considerados os dados de 1.113 mulheres que responderam à questão relativa ao desfecho. A maioria estava na faixa de 40 a 49 anos (24,4%), se autodeclararam brancas (74,6%), 58% tinham até 10 anos de escolaridade e 53% pertenciam à classe C (Tabela 1). Afinal, 199 (17,9%; IC95% 15,6–20,1) relataram ter sofrido VPI em algum momento da vida.

**Tabela 1:** Frequências de violência por parceiro íntimo na vida, segundo das variáveis sociodemográficas, sexuais, reprodutivas, comportamentais e de saúde mental em mulheres adultas (n = 1.113).

Variáveis	n (%)	VPI n (%)	(IC95%)
Idades			
20–29 anos	213 (19)	29 (13)	(9–18)
30–39 anos	240 (22)	33 (14)	(9–18)
40–49 anos	272 (24)	63 (23)	(18–28)
50–59 anos	226 (20)	48 (21)	(16–26)
60–69 anos	162 (15)	26 (16)	(10–21)
Escolaridade			
≥ 15 anos	108 (10)	12 (11)	(5–17)
11–14 anos	359 (32)	45 (12)	(9–16)
5–10 anos	444 (40)	95 (21)	(17–25)
0–4 anos	200 (18)	47 (23)	17–29)
Cor da pele			
Branca	830 (75)	146 (18)	(15–20)
Amarela, parda, indígena, preta	283 (25)	53 (19)	(14–23)
Classe econômica			
A e B	386 (35)	39 (10)	(7–13)
C	587 (53)	120 (20)	(17–23)
D e E	134 (12)	40 (30)	(22–37)
Situação conjugal			
Casadas/em união estável	402 (64)	113 (16)	(17–25)
Solteiras/divorciadas/viúvas	711 (36)	86 (21)	(13–18)
Número de gestações			
Nenhuma	170 (15)	14 (8)	(4–12)
Uma	251 (23)	33 (13)	(9–17)
Duas	265 (24)	39 (15)	(10–19)
Três ou mais	427 (38)	113 (26)	(22–30)
Idade da primeira gestação			
≥ 18 anos	716 (76)	126 (18)	(14–20)
Até 17 anos	224 (24)	57 (25)	(19–31)
Aborto na vida			
Não	1014 (91)	172 (17)	(14–19)
Sim	95 (9)	27 (28)	(19–37)
IST na vida			
Não	1032 (93)	175 (17)	(14–19)
Sim	79 (7)	24 (30)	(20–40)
Número de consultas			
Nenhuma	174 (15)	24 (14	(8–19)
Uma	176 (16)	31 (18)	(12–23)
Duas ou mais	762 (68)	144 (19)	(16–21)
Consumo excessivo de álcool			
Não	1075 (97)	190 (18)	(15–20)
Sim	32 (3)	7 (22)	(6–37)
Fumo			
Não fumante	653 (59)	91 (14)	(11–16)
Ex-fumante	251 (23)	56 (22)	(17–27)
Fumante	203 (18)	52 (26)	(19–31)
Uso de drogas ilícitas na vida			
Não	983 (88)	172 (17)	(15–20)
Sim	130 (12)	27 (21)	(13–27)
Transtorno mental comum			
< 7	670 (60)	84 (12)	(10–15)
≥ 7	443 (40)	115 (26)	(22–30)

IC95%: intervalo de confiança de 95%; VPI: violência por parceiro íntimo; IST: infecções sexualmente transmissíveis.

Na análise bruta, apresentaram associação com o desfecho de VPI, as seguintes variáveis: menor classe econômica, pertencer às classes econômicas C, D e E, ser casada ou estar em união estável, ter tido IST na vida, idade da primeira gestação até 17 anos, ser fumante ou ex-fumante e presença de TMC (Tabela 2). Verificou-se que, após a análise ajustada, menor classe econômica, ter companheiro, ter tido IST na vida e a presença de TMC continuaram apresentando relação com o desfecho. As mulheres pertencentes à classe D/E e que informaram ter tido IST tiveram em torno de duas vezes mais probabilidade do desfecho, enquanto as mulheres com TMC apresentaram 50% mais probabilidade de VPI comparadas às sem TMC.

**Tabela 2:** Razões de prevalência brutas e ajustadas de violência por parceiro íntimo na vida, segundo das variáveis sociodemográficas, sexuais, reprodutivas, comportamentais e de saúde mental em mulheres adultas (n = 1.113).

Variável	Análise bruta		Análise ajustada	
	RP (IC95%)	p	RP (IC95%)	p
Idades^a^		0,063		
20–29 anos	1,00			
30–39 anos	1,01 (0,63–1,60)			
40–49 anos	1,70 (1,14–2,54)			
50–59 anos	1,56 (1,02–2,38)			
60–69 anos	1,18 (0,72–1,92)			
Escolaridade^a^		< 0,001		
≥ 15 anos	1,00			
11–14 anos	1,13 (0,62–2,05)			
5–10 anos	1,92 (1,10–3,38)			
0–4 anos	2,11 (1,17–3,81)			
Cor da pele^a^		0,665		
Branca	1,00			
Amarela, parda, indígena, preta	1,06 (0,80–1,41)			
Classe econômica^a^		**< 0,001**		**0,001**
A e B	**1,00**		**1,00**	
C	**2,02 (1,44–2,84)**		**1,62 (1,15–2,27)**	
D e E	**2,95 (1,99–4,39)**		**1,94 (1,29–2,93)**	
Situação conjugal^a^		**0,021**		0,059
Casadas/em união estável	**1,00**		1,00	
Solteiras/divorciadas/viúvas	**1,35 (1,04–1,73)**		1,29 (1,00–1,66)	
Número de gestações^b^		< 0,001		< 0,001
Nenhuma	1,00		1,00	
Uma	1,60 (0,88–2,89)		1,48 (0,81–2,71)	
Duas	1,79 (1,00–3,19)		1,76 (0,98–3,18)	
Três ou mais	3,21 (1,90–5,44)		2,40 (1,39–4,16)	
Idade da primeira gestação^b^		0,008		
≥ 18 anos	1,00			
Até 17 anos	1,45 (1,10–1,90)			
Aborto na vida^b^		0,004		
Não	1,00			
Sim	1,68 (1,18–2,37)			
IST na vida^b^		**0,002**		**0,002**
Não	**1,00**		**1,00**	
Sim	**1,79 (1,25–2,57)**		**1,70 (1,19–2,42)**	
Número de consultas^b^		0,126		0,119
Nenhuma	1,00		1,00	
Uma	1,28 (0,78–2,08)		1,54 (0,95–2,49)	
Duas ou mais	1,37 (0,92–2,04)		1,41 (0,95–2,09)	
Consumo excessivo de álcool^b^		0,531		
Não	1,00			
Sim	1,24 (0,63–2,41)			
Fumo^b^		**< 0,001**		0,031
Não fumante	**1,00**		1,00	
Ex-fumante	**1,60 (1,19–2,16)**		1,30 (0,97–1,75)	
Fumante	**1,84 (1,36–2,49)**		1,33 (0,98–1,81)	
Uso de drogas ilícitas na vida^b^		0,354		
Não	1,00			
Sim	1,19 (0,83–1,71)			
Transtorno mental comum^b^		**< 0,001**		**< 0,001**
< 7	**1,00**		**1,00**	
≥ 7	**2,07 (1,60–2,67)**		**1,71 (1,32–2,21)**	

IC95%: intervalo de confiança de 95%; RP: razão de prevalência; IST: infecções sexualmente transmissíveis.

Nota: valores em negrito: p < 0,05 e IC95% com efeito.

^a^ Bloco de variáveis sociodemográficas.

^b^ Bloco de variáveis comportamentais, sexuais, reprodutivas e de saúde mental. Cada variável foi ajustada às demais no mesmo nível ou em nível anterior em um modelo hierárquico de causalidade. Apenas as variáveis associadas ao desfecho com p < 0,20 no modelo não ajustado foram posteriormente inseridas e retidas no modelo final ajustado multivariado.

A análise estratificada por faixas etárias objetivou verificar possíveis aspectos geracionais relacionados ao desfecho. Entre as mulheres entre 20 e 39 anos a prevalência de VPI na vida foi de 13,7% (IC95% 10,5–16,8) e para aquelas entre 40 ou mais foi de 20,7% (IC95% 17,6–23,8). Mulheres entre 20 e 39 anos que tinham tido três ou mais gestações, realizaram aborto, eram fumantes ou ex-fumantes e tinham TMC tiveram maiores prevalências do desfecho na análise bruta (Tabela 3). Na análise ajustada, apenas a presença de TMC continuou associada, sendo que essas mulheres apresentaram 2,33 mais probabilidades de já terem sofrido VPI.

**Tabela 3:** Razões de prevalência brutas e ajustadas de violência por parceiro íntimo na vida, segundo das variáveis sociodemográficas, sexuais, reprodutivas, comportamentais e de saúde mental em mulheres adultas, estratificadas por gerações etárias, 20 a 39 anos, 40 anos ou mais (n = 1.113).

Variáveis	20 a 39 anos				40 anos ou mais			
	Análise Bruta		Análise Ajustada		Análise Bruta		Análise Ajustada	
	RP (IC95%)	p	RP (IC95%)	p	RP (IC95%)	p	RP (IC95%)	p
Escolaridade^a^		0,014		0,043		0,017		
≥ 15 anos	1,00		1,00		1,00			
11 a 14 anos	1,07 (0,42–2,73)		1,06 (0,42–2,63)		1,23 (0,56–2,68)			
5 a 10 anos	2,19 (0,90–5,33)		1,94 (0,80–4,70)		1,69 (0,82–3,48)			
0 a 4 anos	1,63 (0,47–5,59)		1,46 (0,43–4,92)		1,88 (0,90–3,93)			
Cor da pele^a^		0,753				0,430		
Branca	1,00				1,00			
Amarela, parda, indígena, preta	0,92 (0,53–1,58)				1,14 (0,82–1,59)			
Classe econômica^a^		0,038				**< 0,001**		**< 0,001**
A e B	1,00				**1,00**		**1,00**	
C	1,64 (0,93–2,89)				**2,25 (1,48–3,44)**		**1,76 (1,15–2,69)**	
D e E	1,96 (0,94–4,11)				**3,55 (2,21–5,69)**		**2,30 (1,42–3,75)**	
Situação conjugal^a^		0,199				**< 0,001**		**0,009**
Casadas/em união estável	1,00				**1,00**		**1,00**	
Solteiras/divorciadas/viúvas	0,71(0,43–1,19)				**1,76 (1,31–2,36)**		**1,46 (1,09–1,95)**	
Número de gestações^b^		**0,002**				< 0,001		0,008
Nenhuma	**1,00**				1,00		1,00	
Uma	**2,10 (1,00–4,40)**				0,89 (0,33–2,41)		0,84 (0,31–2,25)	
Duas	**1,72 (0,71–4,15)**				1,29 (0,53–3,14)		1,30 (0,54–3,14)	
Três ou mais	**3,18 (1,53–6,61)**				2,27 (0,98–5,27)		1,71 (0,73–3,97)	
Idade da primeira gestação^b^		0,223				**0,003**		
≥ 18 anos	1,00				**1,00**			
Até 17 anos	1,36 (0,83–2,24)				**1,64 (1,18–2,28)**			
Aborto na vida^b^		**0,024**				0,081		
Não	**1,00**				1,00			
Sim	**2,17 (1,10–4,27)**				1,43 (0,96–2,15)			
IST na vida^b^		0,183				**0,001**		**0,001**
Não	1,00				**1,00**		**1,00**	
Sim	1,57 (0,81–3,06)				**2,04 (1,35–3,08)**		**1,97 (1,30–2,97)**	
Número de consultas^b^		0,409		0,193		0,386		
Nenhuma	1,00		1,00		1,00			
Uma	1,06 (0,46–2,43)		1,20 (0,53-2,73)		1,30 (0,70–2,41)			
Duas ou mais	1,27 (0,68–2,37)		1,44 (0,79-2,65)		1,30 (0,77–2,20)			
Consumo excessivo de álcool^b^		0,594				**0,018**		
Não	1,00				**1,00**			
Sim	0,69 (0,18–2,65)				**2,24 (1,15–4,35)**			
Fumo^b^		**0,002**				**0,006**		0,203
Não fumante	**1,00**				**1,00**		1,00	
Ex-fumante	**1,95 (1,09–3,46)**				**1,35 (0,95–1,93)**		1,15 (0,83–1,62)	
Fumante	**2,12 (1,24–3,63)**				**1,64 (1,14–2,35)**		1,25 (0,88–1,77)	
Uso de drogas ilícitas na vida^b^		0,159				0,267		
Não	1,00				1,00			
Sim	1,44 (0,87–2,40)				1,36 (0,79–2,36)			
Transtorno mental comum^b^		**< 0,001**		**0,001**		**< 0,001**		**0,006**
< 7	**1,00**		**1,00**		**1,00**		**1,00**	
≥ 7	**2,59 (1,59–4,20)**		**2,33 (1,41–3,87)**		**1,90 (1,42–2,57)**		**1,50 (1,12–2,01)**	

IC95%: intervalo de confiança de 95%; RP: razão de prevalência; IST: infecções sexualmente transmissíveis.

Nota: valores em negrito: p < 0,05 e IC95% com efeito.

^a^ Bloco de variáveis sociodemográficas.

^b^ Bloco de variáveis comportamentais, sexuais, reprodutivas e de saúde mental. Cada variável foi ajustada às demais no mesmo nível ou em nível anterior em um modelo hierárquico de causalidade. Apenas as variáveis associadas ao desfecho com p < 0,20 no modelo não ajustado foram posteriormente inseridas e retidas no modelo final ajustado multivariado.

Entre as mulheres de 40 anos ou mais, associaram-se com a ocorrência do desfecho a menor classe econômica, menor escolaridade, ser solteira, divorciada ou viúva, maior número de gestações, ser mais jovem na primeira gestação, ter tido IST na vida, ter relatado aborto, ser fumante ou ex-fumante, consumo excessivo de álcool e presença de TMC (Tabela 3). Na análise ajustada, continuaram significativas quanto ao desfecho as variáveis classe econômica, situação conjugal, número de gestações, IST, fumo e presença de TMC.

Verificou-se que, nas mulheres de 40 anos ou mais das classes D e E, a probabilidade de VPI foi mais de duas vezes maior em comparação às mulheres da classe A. Ademais, nessas mulheres, solteiras, divorciadas ou viúvas apresentaram 1,45 mais probabilidades de VPI. A ocorrência de IST na vida elevou em quase duas vezes a probabilidade de VPI e as com TMC apresentaram razão de prevalência de 1,50 (Tabela 3).

Análises secundárias exploraram a associação entre VPI e a intersecção das categorias de raça/cor e escolaridade. Para fins de comparação, a Figura mostra as razões de prevalência brutas de VPI para escolaridade e raça/cor separadamente e as prevalências ajustadas para as categorias de intersecção. As razões de prevalência ajustadas do desfecho foram 1,67 maiores (IC95% 1,14–2,45) entre mulheres pretas ou pardas com 10 anos ou menos de escolaridade (n = 178) comparadas às mulheres brancas com 11 anos ou mais de escolaridade (grupo índice; n = 376). Mulheres brancas com menor escolaridade (n = 453) também tiveram maior probabilidade de VPI na vida (RP = 1,58; IC95% 1,14–2,19), enquanto mulheres negras com maior escolaridade (n = 81) evidenciaram menor prevalência do desfecho (RP = 0,67; IC95% 0,31–1,42), embora sem significância estatística.

**Figura:** Razões de prevalência brutas (escolaridade e raça/cor) e ajustadas (categorias interseccionadas) de violência por parceiro íntimo na vida (n = 1.088). Nota: categorias interseccionadas foram ajustadas por idade e situação conjugal.

## DISCUSSÃO

Neste estudo, observou-se elevada prevalência de VPI ao longo da vida entre mulheres adultas residentes na zona urbana de um município do Sul do Brasil, com maior frequência entre aquelas com 40 anos ou mais. A VPI associou-se à menor classe econômica, ao estado civil (ser solteira, divorciada ou viúva), ao relato de IST na vida e à presença de TMC, revelando a interação entre vulnerabilidades sociais, sexuais e psicológicas. Ao estratificar por geração etária emergiram padrões distintos de associação: entre mulheres de 20 a 39 anos, a VPI esteve mais fortemente associada aos TMC, enquanto, entre aquelas com 40 anos ou mais, observou-se maior probabilidade de VPI entre as de classes econômicas mais baixas, com relato de IST e solteiras, viúvas ou divorciadas. Ainda, mulheres pretas ou pardas com menor escolaridade apresentaram maior probabilidade de VPI, o que aponta a influência interseccionada do racismo estrutural e das desigualdades na produção da violência de gênero.

A prevalência encontrada foi semelhante à pesquisa populacional realizada na zona rural do Rio Grande do Sul, onde 17,2% das mulheres relataram violência psicológica ao menos uma vez na vida^16^. Por outro lado, foi inferior à observada em estudo multicêntrico com participação do Brasil, que apontou mais de 45% de violência psicológica, física ou sexual entre mulheres urbanas^2^, bem como à registrada em Maringá (56,0%)^27^. Porém, foi superior a prevalência descrita na Pesquisa Nacional de Saúde de 2019 (7,6% nos últimos 12 meses)^28^.

Estimativas globais de 2000 a 2018 informaram que 27% das mulheres entre 15 e 49 anos sofreram vítimas de violência física ou sexual por parte de um parceiro íntimo, ao menos uma vez na vida^1^. Na América Latina e Caribe, a prevalência de VPI vitalícia foi estimada em 25%, e no Brasil, em 23%^1^ – valores próximos aos deste estudo. Uma metanálise apontou prevalência de 37,3% para qualquer tipo de VPI na vida e de 24,2% no último ano, sendo a violência psicológica a mais comum^29^.

As prevalências de VPI variam amplamente entre estudos nacionais e internacionais, dificultando comparações diretas. Parte dessas diferenças pode se relacionar ao tipo de VPI avaliada e ao período recordatório utilizado. Enquanto a presente pesquisa investigou a VPI ao longo da vida por meio de autorrelato direto, outros inquéritos aplicaram instrumentos padronizados que distinguem tipos de violência e períodos distintos de ocorrência. Dessa forma, é possível que a prevalência aqui aferida esteja subestimada, pois muitas mulheres ainda não reconhecem a violência psicológica, especialmente em suas formas mais sutis, ou a naturalizam^8^. Além disso, variações no contexto urbano e rural, na composição etária das amostras e nos métodos de coleta podem explicar parte das discrepâncias observadas nas prevalências registradas. Ainda assim, o uso de urna, estratégia pouco frequente em inquéritos brasileiros sobre violência, pode ter contribuído para reduzir o viés de desejabilidade social e ampliar a validade das informações autorreferidas.

Neste estudo, a VPI associou-se à menor classe econômica, ser solteira, divorciada ou viúva, relato de IST e presença de TMC. A VPI resulta da interação de fatores em níveis individual, familiar, comunitário e social^1^. Baixo acesso a emprego e renda são reconhecidos como fatores associados à VPI^1,9,11,12,15^ e refletem, em parte, a classe econômica, que neste estudo triplicou a probabilidade de VPI. Resultado semelhante foi encontrado em inquérito nacional na África do Sul, onde mulheres de domicílios com maior índice de riqueza apresentaram menor ocorrência de violência sexual^30^. Assim, a desigualdade econômica – estrutural no sul global — está na origem e na manutenção da VPI^31^. Mulheres mais pobres, com menor instrução e acesso ao emprego remunerado, tendem a depender economicamente do agressor, o que dificulta romper o ciclo da violência^12,30^.

A ocorrência de IST ao longo da vida também se associou à VPI. Embora poucos estudos brasileiros abordem fatores sexuais e comportamentos de saúde vinculados à VPI, nossos achados dialogam com revisões que indicam associação com IST, gestações indesejadas, abortos e sintomas ginecológicos entre mulheres que sofreram violência sexual^32^. Outra revisão apontou alta prevalência de dores pélvicas, ginecológicas e IST entre vítimas de VPI em diferentes países^33^.

Observou-se associação entre presença de TMC e VPI, com mulheres que relataram manifestações de TMC apresentando quase o dobro de probabilidade de referirem VPI. No total da amostra, 39,8% das mulheres apresentaram indicadores de TMC, percentual que aumentou para 57,8% entre aquelas que relataram VPI. O mesmo padrão foi constatado na análise estratificada por faixa etária, com magnitude maior entre mulheres com até 39 anos. Esses achados são consistentes com a literatura, inclusive internacional, que tem demonstrado a forte associação entre VPI e sofrimento psíquico^29,33^.

Evidências indicam que ser vítima de VPI eleva o risco de transtornos mentais e, em algumas condições, essa relação é bidirecional, dada a presença de fatores de risco comuns^35^. Revisões de estudos longitudinais apontam que a depressão pode preceder a violência doméstica, ao passo que sofrer violência aumenta o risco de depressão mesmo em mulheres previamente hígidas^34^. Outro estudo longitudinal australiano com recorte geracional identificou pior saúde mental associada à VPI em todas as idades, com maior impacto na velhice, e mostrou que mulheres com pior saúde mental na juventude estavam mais propensas a sofrer VPI ao longo da vida^17^.

No Brasil, uma coorte prospectiva com 390 mulheres mostrou incidência de 44,6% de TMC nas vítimas de violência nos 12 meses anteriores e de 43,4% nas que relataram violência nos últimos sete anos^36^. Metanálises reforçam essa associação, indicando aumento de três vezes na probabilidade de transtornos depressivos, quatro vezes para transtornos de ansiedade e até sete vezes para transtorno de estresse pós-traumático entre mulheres vítimas de violência^37^. Outra metanálise confirmou a associação da VPI com depressão, TEPT e risco de suicídio^29^. No Reino Unido, um estudo mostrou que 58,4% das mulheres que tentaram suicídio no ano anterior haviam sofrido VPI ao longo da vida^38^.

Diversos estudos, com metodologias distintas, apontaram associação entre VPI e raça/cor^9,10,15^, o que difere dos achados da presente pesquisa. Nossas análises secundárias sugerem que a intersecção entre raça/cor e escolaridade oferece uma via de explicação mais adequada das desigualdades sociais relacionadas à VPI, ao refletir o racismo estrutural e a histórica exclusão das pessoas negras^39^. No estudo, mulheres pretas ou pardas com menor escolaridade tiveram maior probabilidade de VPI do que aquelas nas demais categorias interseccionais (brancas com baixa ou alta escolaridade e negras/pardas com alta escolaridade). Devido ao limitado poder amostral, essa hipótese deve ser testada em estudos futuros com amostragem desenhada para capturar esses entrecruzamentos sociais.

Optou-se por análise estratificada por idade para captar possíveis influências geracionais na associação entre variáveis e VPI – abordagem ainda pouco adotada em estudos sobre o tema. Entre mulheres de 20 a 39 anos, a VPI associou-se à presença de TMC; já entre aquelas com 40 anos ou mais, houve associação com menor classe econômica, estado civil (solteira, viúva ou divorciada), relato de IST e TMC.

Ao contrário dos resultados desta pesquisa, que indicaram maiores prevalências entre as mais velhas, dados de notificações de VPI no Brasil (2011–2017), constataram maiores incidências de VPI entre mulheres de 20 a 39 anos^8^. Já um inquérito nacional espanhol com 8.935 mulheres, que estratificou a prevalência de VPI, fatores associados e impactos da VPI por idade (16–29, 30–49 e ≥ 50 anos), revelou que 15,6% haviam sofrido algum tipo de VPI nos 12 meses anteriores, com maiores taxas entre as mais jovens – o que também contraria nossos achados^18^. Apesar disso, é plausível pensar que, com o avançar da idade, aumenta a exposição ao desfecho, enquanto mulheres mais jovens podem reconhecer, denunciar ou buscar ajuda com mais facilidade, sendo, portanto, mais frequentemente notificadas. Além disso, as diferenças metodológicas entre os estudos que avaliaram a faixa etária e o presente estudo podem explicar essa discrepância.

O estudo espanhol também associou a vulnerabilidade econômica à maior ocorrência de VPI em mulheres de 30 a 49 anos^18^, como na presente pesquisa, pois, entre as mulheres de 40 anos ou mais, as probabilidades de VPI foram duas vezes maiores do que entre as mulheres de classes econômicas mais baixas. Quanto à situação conjugal, observou-se maior probabilidade de VPI entre as solteiras, divorciadas ou viúvas dessa faixa etária, mas não entre as mais jovens. Estudos transversais anteriores já haviam apontado tendência semelhante^40,41^, embora sem estratificar por fase de vida. Uma hipótese para os nossos achados é que mulheres mais velhas podem ter vivenciado relacionamentos abusivos ao longo da vida e quando já se encontravam mais estabelecidas, com os filhos adultos, conseguiram romper o ciclo de violência. Já entre as mulheres mais jovens, a menor prevalência de VPI pode ser explicada pelo menor tempo de exposição.

Ao tratar da VPI, uma limitação recorrente nos estudos envolve o estigma, a vergonha e a culpa decorrentes da cultura patriarcal hegemônica^7,10^. Assim, apesar da alta prevalência encontrada, é possível que os dados estejam subestimados, já que muitas mulheres ocultam ou têm dificuldade em reconhecer as violências sofridas – o que pode ter sido mitigado, neste estudo, pela forma abrangente de avaliação do desfecho. Embora o rastreio populacional da VPI tenha sido facilitado, ao avaliar o desfecho por meio de uma única pergunta, a sensibilidade da mensuração pode ter sido diminuída para os diferentes tipos de violência, atenuando as associações observadas. Outra limitação, inerente ao delineamento transversal, é a impossibilidade de determinar se a VPI é causa ou consequência dos fatores associados. Contudo, o estudo foi conduzido com rigor metodológico, incluindo o uso da urna para assegurar a autenticidade das respostas e garantindo a privacidade durante as entrevistas. Ainda, embora os achados contemplem a situação das mulheres adultas residentes em São Leopoldo no ano de 2015, acredita-se que estes fornecem dados detalhados que podem subsidiar ações de monitoramento da violência contra a mulher.

## CONCLUSÃO

Conjuntamente, os achados indicam a necessidade de políticas públicas intersetoriais voltadas à prevenção e ao enfrentamento da VPI que integrem ações de saúde, assistência social e justiça, com atenção especial às mulheres em maior vulnerabilidade econômica e às faixas etárias mais avançadas. Em especial, sugere-se a importância de fortalecer estratégias que articulem o cuidado em saúde mental e a melhora nas condições socioeconômicas e de proteção social, como preconizado pela Política Nacional de Enfrentamento à Violência contra as Mulheres^42^. Assim, os resultados podem apoiar o planejamento de ações de educação permanente e a capacitação continuada de profissionais de saúde na perspectiva da complexidade social do fenômeno, considerando que os serviços de saúde podem ser procurados por questões de saúde mental que subjazem ou encobrem situações de violência. Entende-se que o fortalecimento de ações de matriciamento, promoção e prevenção em saúde mental na rede de saúde, favoreceria não apenas a identificação e acolhimento de situações de violência, como a construção de estratégias de prevenção e mitigação das repercussões psicológicas da violência de gênero nos territórios cobertos pela atenção primária à saúde.

A partir dos padrões geracionais observados, as abordagens preventivas deveriam considerar as distintas etapas do ciclo de vida e o desdobramento de trajetórias de vulnerabilidade. Ou seja, entre mulheres mais jovens, ações educativas sobre relacionamentos abusivos e saúde mental podem ser mais efetivas, bem como maior atenção a sinais de sofrimento psíquico no âmbito dos cuidados em saúde. Já para mulheres mais velhas seria importante ampliar e fortalecer as políticas de inserção laboral, bem como as redes comunitárias e de suporte social que facilitem o rompimento de vínculos violentos, com ênfase entre aquelas com maior vulnerabilidade socioeconômica.

Por fim, reitera-se a importância da condução de novos estudos sobre o tema, nas diversas realidades, para que se aprimorem políticas públicas, trazendo benefícios para as mulheres que possuem histórico de VPI ou que estão vivenciando essa grave situação. Sugere-se que o monitoramento da VPI, seja em inquéritos populacionais ou nos serviços públicos, contemple indicadores sensíveis às desigualdades de gênero, raça e geração, a fim de orientar políticas de prevenção mais equitativas.

## Data Availability

O banco de dados relacionado a esta pesquisa está disponível sob solicitação à autora de correspondência.
